# Does Double Mean Trouble? Coexistence of Myeloproliferative and Lymphoproliferative Neoplasms

**DOI:** 10.3390/jcm13061816

**Published:** 2024-03-21

**Authors:** Danijela Lekovic, Jelena Ivanovic, Tatjana Terzic, Maja Perunicic Jovanovic, Marija Dencic Fekete, Jelica Jovanovic, Isidora Arsenovic, Vojin Vukovic, Jelena Bila, Andrija Bogdanovic, Darko Antic

**Affiliations:** 1 Clinic of Hematology, University Clinical Center of Serbia, 11000 Belgrade, Serbiamajaperunicicjovanovic@yahoo.com (M.P.J.); jlcjovanovic@yahoo.com (J.J.); vojinvukovic@yahoo.com (V.V.); biladr.jelena@gmail.com (J.B.); andrija.bogdanovic@gmail.com (A.B.); darko.antic1510976@gmail.com (D.A.); 2 Faculty of Medicine, University of Belgrade, 11000 Belgrade, Serbia; 3 Institute of Pathology, Faculty of Medicine, University of Belgrade, 11000 Belgrade, Serbia; tatjana.terzic63@gmail.com (T.T.); marijadfekete@yahoo.com (M.D.F.)

**Keywords:** myeloproliferative neoplasm, lymphoproliferative neoplasm, prognosis, thrombosis, survival

## Abstract

**Background:** The occurrence of myeloproliferative neoplasms (MPNs) that evolve into each other is well-described, as is this occurrence of lymphoproliferative neoplasms (LPNs). However, less is known about rare MPN/LPN coexistence, and the aim of our study was to analyze charachteristics of these patients after long term follow-up. **Methods:** Fourteen patients with MPN/LPN coexistence were diagnosed and treated according to guidelines at a single university center across two decades. **Results:** The overall median age was 53 years (22–69). MPNs patients with subsequent LPNs had a shorter period of second malignancy development and a more aggressive course of LPN, which can cause fatal outcomes. Polycythemia vera and chronic lymphocytic leukemia were most commonly associated (36%). The *JAK2V617F* mutation had 2/3 and cytogenetic abnormalities occurred in 1/3 of patients. MPN/LPN coexistence cases had significantly higher thrombotic potential (42.8%) and a higher third malignancy accruement frequency (21.4%) versus those without such malignancies. **Conclusions:** Considering the younger ages at MPN diagnosis, it is recommended to check regularly for blood lymphocytosis or lymphadenopathy occurrences and organomegaly progression faster than expected for MPN, with the aim of timely LPN diagnoses. The presence of molecular-cytogenetic abnormalities in a majority of patients indicate possible genetic instability and increased risk of development of multiple neoplasms, thus elevating thrombotic risk.

## 1. Introduction

The coexistence of myeloproliferative and lymphoproliferative neoplasms in a single individual is relatively rare. Myeloproliferative neoplasms (MPNs) and lymphoproliferative neoplasms (LPNs) are two distinct categories of blood disorders, and they involve abnormal growth of different types of blood cells.

Myeloproliferative neoplasms primarily affect the myeloid stem cells, leading to the overproduction of mature blood cells, such as red blood cells, white blood cells and platelets. Common types of MPNs include polycythemia vera, essential thrombocythemia and primary myelofibrosis. The MPNs are clonal hematological disorders characterized by increased proliferation of one or more myeloid lineages in the bone marrow (BM) [1,2]. MPNs are slowly progressing diseases which can transform into severe bone marrow failure or acute leukemia.

Lymphoproliferative neoplasms involve the lymphoid stem cells, characterized by uncontrolled production of lymphocytes that can cause monoclonal lymphocytosis, lymphadenopathy, and can infiltrate bone marrow and/or solid organs [3]. Among LPNs, there are indolent as well as aggressive types of the disease. Chronic lymphocytic leukemia usually co-exists with MPNs and LPN, but one of the aggressive lymphomas, such as diffuse large B-cell non-Hodgkin lymphoma (DLBCL), can been also observed in cases. Considering MPNs and LPDs have different pathogenetic mechanisms, it is unclear which cause leads to the appearance of these two malignancies simultaneously. Some studies have shown that risk of developing a second neoplasm was significantly increased in patients with myeloproliferative neoplasms, as was the risk of developing a second hematologic malignancy which affects the quality of life and therefore the overall survival rate of these patients [4,5]. Here we present the characteristics of patients in our group with the coexistence of MPNs and LPNs, with the presentation of two cases in particular being singled out because of the specific course of disease.

## 2. Materials and Methods

Fourteen patients have been identified with both MPNs and LPNs in the Clinic of Hematology, University Clinical Center of Serbia (UCCS) between 2000 and 2022. The overall frequency of LPN was 0.95% of all MPNs patients during the study period. MPN diagnoses were made according to WHO classification at the time of diagnosis. The LPNs were diagnosed according to the actual guideline using blood tests (including blood flow cytometry), physical exams, pathohistological verification and radiographic methods, including CT and NMR scans. In all patients, cytogenetic analyses were done and, when possible, molecular tests were used to identify specific mutations. Standard prognostic models were used for both MPNs and LPNs.

## 3. Results

More than 50% patients received MPNs as a first diagnosis, in contrast to 21.5% of patients with LPNs as first diagnosis (the two diagnoses were also concomitant in 21.5% of patients (Table 1)). Gender distribution was similar, with 57% of males. Patients who had the first appearance of one and then another disease were significantly younger compared to those in whom the disease was presented simultaneously. The median age in the whole group was 53 years (range 22–69). The most common association was between PV and CLL (36%). The most common type of MPN was PV (57%), followed by PMF (33%) and ET (10%), while from LPNs there was CLL in the same representation (57%), then multiple myeloma (21%), DLBCL (15%) and lymphoblastic lymphoma (7%). The period until the development of LPNs after the initial diagnosis of MPNs is twice as short, and the average time is 52 months, in contrast to the development of LPNs followed by MPNs, which is 101 months. Analyzing the frequency of the cytogenetic−molecular markers, 2/3 of the patients exhibited the presence of *JAK2V61F* mutations, while 1/3 had cytogenetic aberrations, most often in LPNs alone, then followed by coexistence of these two diseases. Overall, 57% of MPN patients required a myeloid-specific treatment, while patients with LPNs were treated in 64.3% of patients. Nearly half of patients (42.8%) developed thrombotic complications in the whole group, but this occurred more often in MPNs followed by LPNs. An interesting observation of note is the development of a third malignancy in 21.4% of patients. The median follow-up was 7.5 years in the whole group, and a median survival of 12.5 years was reached in the group of MPNs followed by LPN development. During follow-up treatment, 35% of patients died due to the progression of LPDs (two patients), heart failure (one patient), renal failure (one patient) and COVID-19 (one patient).

### 3.1. Case Report 1

**Figure 1:**
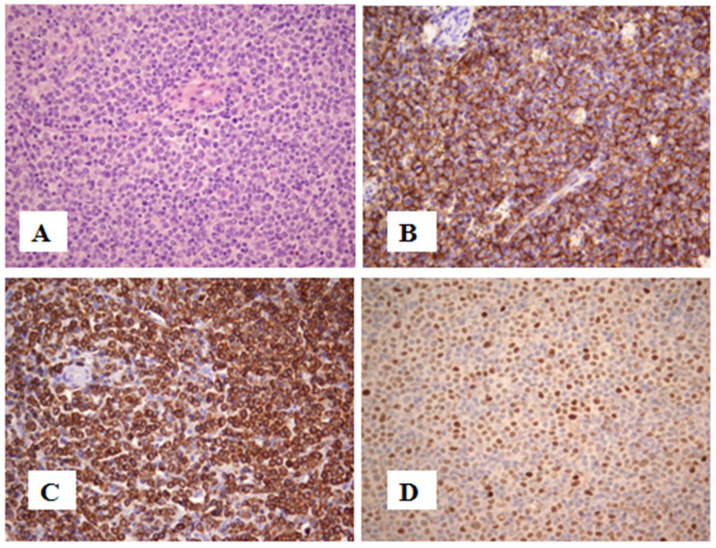

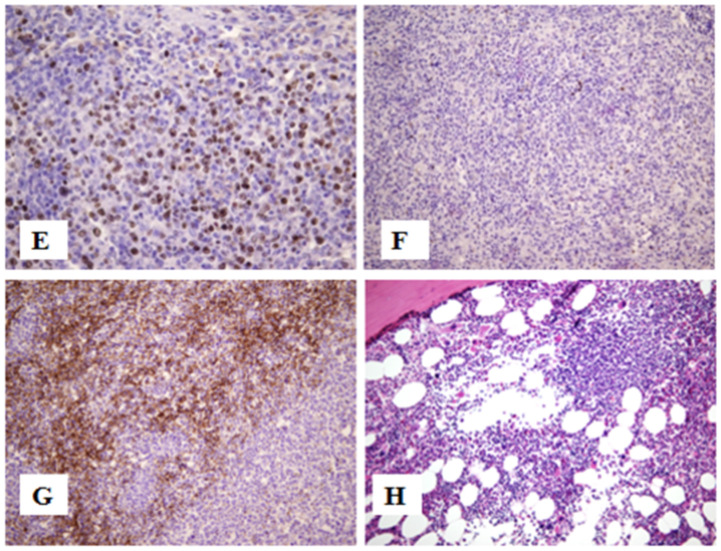
(**A**): Spleen diffusely infiltrated by DLBCL (HE, ×400). Immunohistochemical stains demonstrated the neoplastic cells expressing in spleen (**B**): CD20, (**C**): bcl-2, (**D**): c-myc, (**E**): Ki-67+ 40%, (**F**): Rare CD71+ erythroblasts in the sinusoids of the spleen. (Immunoperoxidase, ×400); (**G**): CD61+ platelet aggregates without visible megakaryocytes in sinusoids of the spleen (Immunoperoxidase ×200); (**H**): Bone marrow biopsy showed morphology of early fibrotic PMF associated with 30% infiltration of small lymphocytes (HE, ×200).

**Figure 2:**
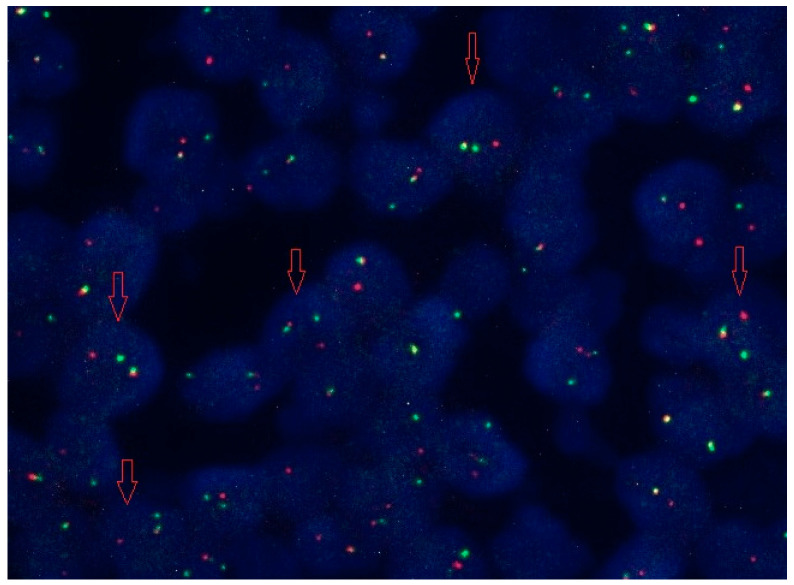
Fluorescence in situ hybridization showed BCL-2+ rearrangement (red arrows indicate positive nuclei).

A 55-year-old female patient was admitted to the Clinic of Hematology, UCCS, in 2015 due to persistent thrombocytosis and mild splenomegaly. The patient reported no other symptoms. In her medical history, she had ventricular arrhythmias upon physical examination and was an active smoker. A physical examination revealed splenomegaly (+2 cm) below the costal margin, which was confirmed by ultrasonography (14.6 × 5.5 cm). The initial complete blood cell (CBC) count showed a white blood cell (WBC) level of 11.8 × 10^9^/L, with 65% neutrophils, 26% lymphocytes, 3% eosinophils, 5% monocytes and 1% basophils in formula, hemoglobin (Hb) level of 149 g/L and platelet (Plt) count of 656 × 10^9^/L. The results of biochemical analyses were normal, except elevated serum lactate dehydrogenase (LDH) levels of 400 U/L (150–320). The *JAK2V617F* mutation was detected by PCR analysis. Conventional cytogenetics showed a normal female karyotype. Spirometry was normal, as was the concentration of serum erythropoietin. A bone marrow (BM) biopsy showed features compatible with a primary myelofibrosis (PMF), prefibrotic stage with fibrosis grade 1. The patient was diagnosed with prefibrotic PMF (prePMF) according to the 2016 revision of the World Health Organization (WHO) classification. According to the International Prognostic Scoring System (IPSS) score of 1, the patient was classified as low risk and, furthermore, was regularly followed every three months. She was only treated with acetylsalicylic acid as an antithrombotic prophylaxis due to arrhythmias. In September 2021, the patient’s spleen size by ultrasound was 16 cm. In the regional hospital, due to stomach pain in March 2022, a computer tomography (CT) scan was done which showed progressive splenomegaly, measuring 26 × 10 cm, with necrosis segments; therefore, an urgent splenectomy was performed. Before the splenectomy, a CBC count showed mild anemia (Hb 111 g/L), with normal WBC (7.2 × 10^9^/L) and a normal Plt count (380 × 10^9^/L). Initial histopathological examination of the spleen resulted in a diagnosis of marginal zone lymphoma. In April 2022, she had a SARS-CoV-2 infection that was intensive, and she was hospitalized in a COVID-19 hospital and treated with Sotrovimab. Due to progression of thrombocytosis with a Plt count of 1500 × 10^9^/L, cytoreductive therapy was started with Hydroxyurea (HU) at 3 × 500 mg per day, and after recovery from the SARS-CoV-2 infection she was referred to our clinic for a re-evaluation in July 2022. The pathohistological revision of the spleen with immunohistochemistry was done at the Faculty of Medicine, University of Belgrade, and the final diagnosis of Diffuse large B cell lymphoma, NOS (DLBCL), double expressor (DEL) was confirmed with very rare extramedullary hematopoiesis (EMH) signs (Figure 1G). In July 2022, the bone marrow biopsy showed MPNs associated with 30% infiltration of small lymphocytes (Figure 1H). The Ki67 proliferative index was approximately 40%. Fluorescence in situ hybridization (FISH) analysis confirmed only a BCL-2+ rearrangement (Figure 2). A Computer Tomography (CT) scan of the neck, thorax, abdomen and pelvis showed an axillary lymphadenopathy less than 2 cm. The initiation of specific hematological treatment was indicated, but the patient did not have all the necessary vaccinations after her splenectomy. Before vaccination was done, she had a SARS-CoV-2 re-infection, and the hospitalization was postponed again. In the meantime, the patient’s general condition worsened, her performance status was 3/4, in terms of her blood count, and she had leukocytosis with a WBC of 32 × 10^9^/L and a normal range of hemoglobin and platelet levels. Her LDH levels were extremely elevated, and hypoalbuminemia was observed. A CT body scan showed further lymphoma progression with generalized lymphadenopathy and bilateral pleural effusions. In October 2022, at a regional hospital, treatment with a CHOP protocol (cyclophosphamide, doxorubicin, vincristine, prednisolone) was started due to vital indications. After three courses of CHOP chemotherapy, control scans showed further lymphoma progression, now staged as CS IV B E M+ according to the Ann Arbor staging. Despite all therapeutic measures, no clinical improvement was obtained, and the patient died due to sepsis.

### 3.2. Case Report 2

**Figure 3:**
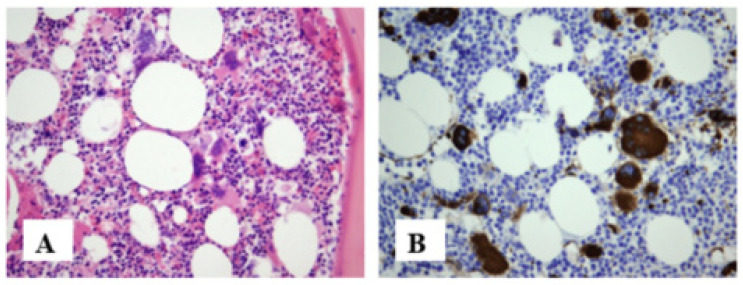

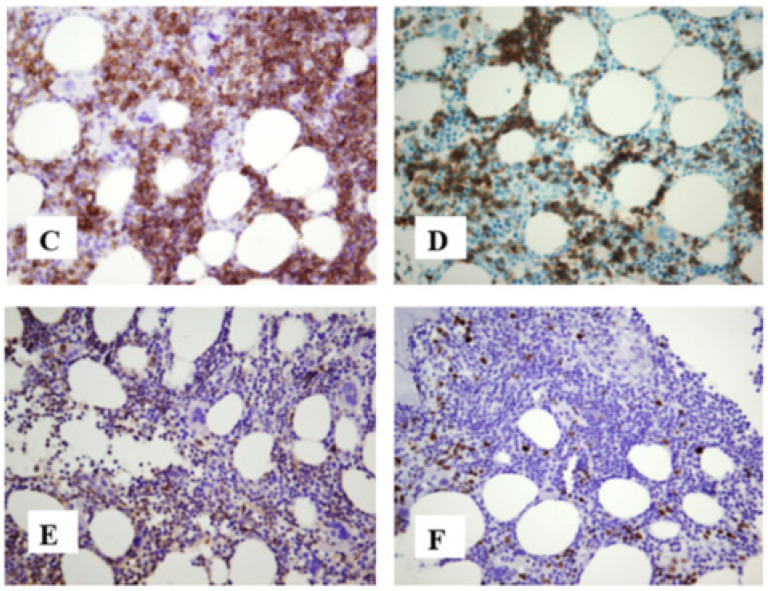
(**A**): bone marrow biopsy (HE, ×400) (March 2015) showed morphology of pre PMF, without lymphocytic infiltration; (**B**): Immunohistochemical stains demonstrated CD61+ polymorphic and atypical megakaryocytes with uneven distribution and clustering (Immunoperoxidase, ×400). (**C**): Bone marrow biopsy (February 2020) demonstrated interstitial infiltration of small lymphocytes, showing immunohistochemical staining for CD20, (**D**): CD5, (**E**): CD38, (**F**): Ki67+ in 10% of lymphoid cells (Immunoperoxidase, ×400).

In March 2015, a 57-year-old female with a history of arterial hypertension and unstable pectoral angina was referred to a hematologist due to an unexplained persistent increase in platelet count over almost two years. Physical examination was unremarkable, with no organomegaly or enlarged lymph nodes. Her blood analysis showed a Hb of 139 g/L, thrombocytosis of 728 × 10^9^/L, WBC of 20.8 × 10^9^/L, with neutrophil predominance (72%) and mild elevated LDH of 506 U/L (220–460). A chest X-ray and abdominal ultrasound showed no pathological findings. Karyotype was normal. Mutation of V617F in the JAK2 gene was not observed by PCR analysis. BM biopsy was performed in another medical center and showed hypercellularity, polymorphic and dysplastic megakaryocytes, with uneven distribution and clustering (Figure 3A,B). According to WHO criteria, a diagnosis of prePMF was made. She was stratified as intermediate-1 risk group and treated with cytoreductive therapy (HU 1 g/daily), with complete and sustained remission. However, in April 2018, three years following PMF diagnosis and HU treatment, progressive leukocytosis (28 × 10^9^/L) with lymphocytosis (83%) was observed; therefore flow cytometry immunophenotyping of peripheral blood was performed, which showed expression of CD5, CD23, in combination with dim CD20, CD22 and CD43, with dim monoclonal surface immunoglobulin and negative CD38 and CD49b. According to the immunophenotypic characteristics of clonal B lymphocytes, the Matutes CLL score was highest (5 points). A repeated BM biopsy at that time showed 60% of CD20+, CD5+, CD23+ infiltrative cells, with 10% Ki-67 positive cells (Figure 3C,D). These findings were consistent with chronic lymphocytic leukemia (CLL). Our patient was initially managed with a “watch and wait” approach. Two years later, during the follow-up, she developed B symptoms, clinically significant lymphadenopathy (max diameter 4 × 2 cm), as well as progressive leukocytosis (WBC 150 × 10^9^/L) and lymphocytosis (absolute lymphocyte count, ALC 133.5 × 10^9^/L) and a lymphocyte doubling time ˂ 6 months, which fulfilled the criteria for treatment initiation. Fluorescence in situ hybridization (FISH) did not show any negative molecular prognostic markers, and repeated cytogenetic analysis indicated normal karyotypes. In our patient, a diagnosis of CLL Rai stage II; Binet stage B was made, with a cumulative index rating scale (CIRS) of 6. In August 2020, treatment with Obinutuzumab−chlorambucil therapy was started. After completing six consecutive immunochemotherapy cycles, complete remission of CLL was achieved, and it was held until today. In April 2022, the CBC count showed a normal Hb level and normal WBC, but progression of thrombocytosis up to 727 × 10^9^/L and treatment with hydroxyurea 500 mg per day was started.

## 4. Discussion

Occurrence of two distinct hematology neoplasms in the same patient is very rare; therefore, little is known in regard to the clinical characteristics, thrombotic complications and survival chances of this scenario. Considered etiopathogenetic mechanisms, some genetic abnormalities (*JAK2V617F*, *BCR/ABL1*, *PDGFRA*, *PDGFRB*, *TET2*, *SF3B1*) were described which can lead to either myeloid or lymphoid malignancies [6,7,8]. A Swedish registry study revealed a 5- to 7-fold elevated risk of MPNs and a 1.6-fold increased risk of CLL among first-degree relatives of MPN patients [9]. The fact that family members of individuals with MPNs are at higher risk for the development of MPNs and CLL suggests that host genetic modifiers also have a role in the pathogenesis of these malignancies. A study of Le Bousse-Kerdilès MC showed that, in PMF, clonal cells produce inflammatory cytokines, and chronic inflammation promotes specific B-cell tumorigenesis by providing an environment where neoplastic B cells escape normal regulatory mechanisms [10]. Chronic inflammation is an accompanying manifestation of all types of MPNs, and this may explain the higher rate of prior MPNs over LPNs in our study. External triggers, such as hematological therapies (hydroxyurea, ruxolitinib), may also favor the emergence of the second clone by reducing immune surveillance upon the development of second tumors. Interest in the potential role of drugs in lymphomagenesis has been strengthened after the publication of an Austrian study [11] that raised the possibility that the JAK inhibitor, ruxolitinib, and treatment for myelofibrosis were associated with an increased risk for aggressive B-cell lymphomas [12,13]. In 2019, the European Leukemia Network conducted an international case–control study, including 1881 patients with MPN, with the aim of analyzing the risk of developing secondary cancer after exposure to cytoreductive drugs, including hydroxyurea and ruxolitinib, as well as a combination of drugs [14]. The results of the study did not show an increased risk of secondary hematological cancer and cancer development compared to unexposed patients, while all of these drugs were associated with an excess risk of non-melanoma skin cancer. *Porpaczy* et al., in a recent real-world data study conducted in Italy including 219 patients with myelofibrosis treated with ruxolitinib, did not register a case of developing lymphoma, although the median follow-up after initiation of ruxolitinib matched the median time to lymphoma onset [15]. None of the patients in our study received JAK2 inhibitor ruxolitinib. In our study group, the frequency of MPN and LPN treatment is not higher than if these patients were treated for only one disease. Based on the above, in our group of patients, no connection was observed between the use of therapy and the development of a secondary malignancy.

According to our results, the median age of the patients was less than 60 years, and the youngest were in a group that first developed LPNs and then MPNs (49 years). More than 50% of patients had firstly developed MPNs before developing LPNs after a twice shorter time than vice versa. One third of patients had a combination of PV and CLL. Median survival was only reached in a group of MPNs, then LPNs, and it was 196 months. On the other hand, in a systematic review by Marchetti et al., 214 patients were included that harbored both MPNs and LPDs, diagnosed and treated from 2005–2016 [16]. The median age of the patients in the study was older than in our group (67 years). Distribution of patients according to the type of MPN was different, covering mostly patients with essential thrombocythemia (44%) then polycythemia vera (29%), primary myelofibrosis (23%) and unclassified MPN (4%). In this study, patients more often developed LPNs after MPNs, but after a longer median time of 72 months. The median survival after MPN diagnosis was much shorter than in our group (96 months vs. 196 months). In our study, median survival is longer for the group of patients who were first diagnosed with MPNs and then LPNs compared to other studies, probably due to age at diagnosis (younger population) and the cause of death being more frequently due to comorbidities than from active hematological disease. In the previously mentioned study, the patients were older and developed more often aggressive forms of LPNs. 

According to the work of Vannucchi et al., patients with the coexistence of MPNs and LPNs were observed in 1.34% of all MPN patients during the study period, and these patients were older with a shorter follow-up period [17]. This study included only patients with PV and ET [17], which is in contrast to our group of patients, where the incidence of LPN was 0.95% of all MPN patients during the study period, featuring a lower frequency than in the previously described study, probably due to the inclusion of patients with PMF and an increase in analyzed MPN populations in that case.

Splenic infiltration is often seen in DLBCL, but the type of primary splenic DLBCL is rare and studies on its clinicopathological features are limited. Holst J et al. evaluate patients with confirmed dual diagnoses of myeloproliferative neoplasm and lymphoma [18]. They found that the 5-year overall survival rate of the patients with MPN + DLBCL was 19%, as compared to 34% for patients in the matched reference cohort with a presence of DLBCL only. Also, the 5-year overall survival of the patients with MPN + CLL was 65% compared to 79% in patients with only CLL. Another study compared 66 patients with primary splenic DLBCL and 309 patients with DLBCL NOS [19]. They found that primary splenic DLBCL had a more favorable progression free survival (PFS) compared to the DLBCL control, but there is no difference in overall survival between the above-mentioned groups. It is interesting that in our first case we were looking for a sign of EMH in the spleen after splenectomy, which is typically a cause of organomegaly and is a relatively common complication of PMF; however, although a BM biopsy showed the presence of a myeloproliferative neoplasm associated with 30% infiltration of small lymphocytes, the histopathology of the spleen has shown an absence of EMH signs. Instead of that, the diagnosis was primary splenic DLBCL. Furthermore, DLBCL is an aggressive tumor in itself, and poor clinical outcome is increased by overexpression of MYC and BCL2 proteins (double-expressor lymphoma, DEL) and a post-germinal immunohistochemical subtype of DLBCL (non-germinal center B-cell (non-GCB)-DLBCL) [20]. Both of these two unfavorable prognostic factors were present in our first described patient, which led to rapid disease progression despite low initial risk. The patient had limited treatment efficacy with CHOP chemotherapy. The delay in necessary vaccinations and subsequent SARS-CoV-2 reinfection likely contributed to the patient’s compromised immune status, exacerbating the disease progression treatment complication with sepsis and shorter survival. 

The second case described showed development of CLL in patients with prePMF. The patient’s response to Obinutuzumab−chlorambucil therapy for CLL was successful, leading to complete remission and sustained improvement. The absence of negative molecular prognostic markers in FISH and normal karyotype indicates a relatively favorable prognosis for the CLL. Despite successful CLL treatment, the patient experienced a progression of thrombocytosis, requiring the initiation of hydroxyurea therapy, suggesting the challenges in managing multiple hematological conditions concurrently. These presented cases indicate that the course of both diseases depends a lot on the presence of poor prognostic parameters previously defined for the diseases themselves. Laurenti L. et al. evaluated patients with confirmed dual diagnoses of MPN and CLL, and they found that MPN therapy does not interfere with the prognosis of patients with CLL [21]. Burgstaller S et al. showed lenalidomide was effective in patients diagnosed with a myeloproliferative and a lymphoproliferative disease at the same time [22].

The limitation of our study is the small sample of patients; however, some important observations were seen, and these patients may have an unusual appearance and course of the disease. We have described the development of a rarity such as diagnosed diffuse B-cell lymphoma in the spleen of a patient with MPN where extramedullary hematopoiesis is usually expected. Also, the course of chronic lymphocytic leukemia (CLL) was more aggressive than expected, although, in this case, there were no unfavorable prognostic parameters at diagnosis. This is particularly characteristic of the group who first developed MPNs then LPNs. This group of patients also have a shorter period between two malignancies and a more aggressive course of LPNs, which can cause fatal outcomes. However, some important observations were also seen further to unusual presentation, such as the importance of assessment of prognostic factors, as nearly half of patients (42.8%) developed thrombotic complications. The thrombotic potential was higher than previously described in the study of Bucelli et al. [13], where it was 16%. A potential explanation could be the increased risk of development of a third malignancy, which was seen in 1/5 of patients. Activation of the coagulation system is significantly increased in the presence of two or even more malignancies, and there is also a thrombotic risk due to this [23]. Increased thrombotic risk could be in relation also with *JAK2V617F* mutation, which was detected in 66.7% of patients. A third observation is the higher frequency of cytogenetic abnormities seen in 1/3 of patients, indicating possible genetic instability and an increased risk of the development of multiple neoplasms [24,25,26].

In conclusion, considering the younger age rate at the diagnosis of MPN, it is advised to pay attention to lymphocytosis development in blood as well as to the development of lymphadenopathy or the progression of organomegaly faster than expected for MPNs, with the aim of timely diagnosis of LPNs. Additionally, higher thrombotic risk indicates prioritizing measures, such as antithrombotic prophylaxis, to prevent complications and improve the outcomes of these patients. Due to possible genetic instability, these cases underscore the importance of timely and accurate diagnosis following regular check-up and comprehensive patient care management because of higher risks of development, as well as the third malignancy. Controlled studies are needed to better refine individuals at higher risk of developing second hematological malignancies and the possibility of deeper molecular analyses, including Next Generation Sequencing (NGS) assays, in these patients.

## Figures and Tables

**Table 1:** Characteristics of patients according to time of MPNs and LPNs diagnosis.

	MPNs ^1^ as First Diagnosis	MPNs + LPNs Co-Occurrence	LPNs ^2^ as First Diagnosis	Total
Number of patients	8 (57%)	3 (21.5%)	3 (21.5%)	14 (100%)
Age at diagnosis (years)	52 (22–63)	59 (42–69)	49 (41–60)	53 (22–69)
Male	4/8	3/3	1/3	8/14 (57%)
First and second diagnosis	1 PV ^3^/NHL DLBCL ^4^ 1 PMF ^5^/NHL DLBCL ^4^ 1 PMF ^5^/CLL ^6^ 1 ET/CLL ^6^ 1 PV ^3^/CLL ^6^ 1 ET ^8^/MM ^7^ 1 PV ^3^/MM ^7^ 1 PV ^3^/lymphoblastic lymphoma	2 CLL ^6^ + PV ^3^ 1 MM ^7^ + PMF ^5^	2 CLL ^6^/PV ^3^ 1 atypical CLL ^6^ (score 3)/PMF ^5^	5 PV ^3^ + CLL ^6^ 2 PMF ^5^ + CLL ^6^ 1 ET ^8^ + CLL ^6^ 1 PMF ^5^ + MM ^7^ 1 ET ^8^ + MM ^7^ 1 PV ^3^ + MM ^7^ 1 PMF ^5^ + NHL DLBCL ^4^ 1 PV ^3^ + NHL DLBCL ^4^ 1 PV ^3^ + lymphoblastic lymphoma
Time before second diagnosis (months)	Until the appearance LPDs: 52		Until the appearance MPNs: 101	65 (4–216)
*JAK2V617F* + mutation (MPNs ^1^) CG aberration ^12^ (LPNs ^2^)	4/7 (57%) 1/8 (12.5%)	2/2 (100%) 1/3 (33.3%)	2/3 (66.7%) 2/3 (66.7%)	8/12 (66.7%) 4/14 (28.5%)
Chemotherapy in MPNs LPNs ^2^	^1^ HU ^9^ 6/8 (75%) 5/8 (62.5%)	HU ^9^ 1/3 (33.3%) 2/3 (66.7%)	HU ^9^ 1/3 (33.3%) 2/3 (66.7%)	8/14 (57%) 9/14 (64.3%)
Third malignancy	1/8 (12.5%)	2/3 (66.7%)	0/3 (0%)	3/14 (21.4%)
Thrombosis	4/8 (50%)	1/3 (33.3%)	1/3 (33.3%)	6/14 (42.8%)
Follow-up median (months) Median OS ^13^ (months)	99 (36–252) 196 (127–251)	60 (26–84) Not reached	101 (74–174) Not reached	90 (26–252) 200 (149–251)
Cause of death	4/8 (50%) 2 LPNs ^2^, 1 ARF ^10^, 1 HF ^11^	1/3 (33.3%) COVID	Alive	5/14 (35.7%)

^1^ Myeloproliferative neoplasms—MPNs; ^2^ Lymphoproliferative neoplasms—LPNs; ^3^ Polycythemia vera—PV, ^4^ Difuse large B cell non Hodgkin Lymphoma—NHL DLBCL; ^5^ Primary Myelofibrosis—PMF; ^6^ Chronic Lymphocytic Leukemia—CLL; ^7^ Multiple myeloma—MM; ^8^ Essential Thrombocythemia—ET; ^9^ HU—hydroxyurea; ^10^ Acute Renal Failure—ARF; ^11^ Heart failure—HF; ^12^ CG- cytogenetic (aberration analyzed by convetional cytogenetic); ^13^ Overall survival.

## Data Availability

All data regarding this research are available upon reasonable request to the corresponding author.
